# Stoichiometry of C:N:P in the Roots of *Alhagi sparsifolia* Is More Sensitive to Soil Nutrients Than Aboveground Organs

**DOI:** 10.3389/fpls.2021.698961

**Published:** 2021-10-12

**Authors:** Hui Yin, Hongwei Zheng, Bo Zhang, Akash Tariq, Guanghui Lv, Fanjiang Zeng, Corina Graciano

**Affiliations:** ^1^College of Resource and Environment Sciences, Xinjiang University, Urumqi, China; ^2^State Key Laboratory of Desert and Oasis Ecology, Xinjiang Institute of Ecology and Geography, Chinese Academy of Sciences, Urumqi, China; ^3^University of Chinese Academy of Sciences, Beijing, China; ^4^Xinjiang Key Laboratory of Desert Plant Roots Ecology and Vegetation Restoration, Xinjiang Institute of Ecology and Geography, Chinese Academy of Sciences, Urumqi, China; ^5^Cele National Station of Observation and Research for Desert-Grassland Ecosystems, Cele, China; ^6^Research Center for Ecology and Environment of Central Asia, Chinese Academy of Sciences, Urumqi, China; ^7^Instituto de Fisiología Vegetal, Consejo Nacional de Investigaciones Científicas y Técnicas, Universidad Nacional de La Plata, Buenos Aires, Argentina

**Keywords:** desert plant, ecological stoichiometry, leguminous plant, nutrients, water stress, soil depths

## Abstract

The stoichiometry of carbon, nitrogen, and phosphorus (C:N:P) among leaves, stems, and roots reflects trade-offs in plants for acquiring resources and their growth strategy. The widely distributed plant *Alhagi sparsifolia* is an ideal species to study the ecological stoichiometry in different organs in response to the availability of nutrients and water in the desert ecosystem. However, which response of organs is most sensitive to environmental conditions is still unclear. To answer this question, we collected samples of plants and soils including not only aboveground leaves and stems, but also underground roots and soils from a wide range of arid areas during the growing season. The C, N, P, C:N, C:P, and N:P ratios in leaves, thorns, stems, and roots were derived to explore their relationship as well as their response mechanisms to nutrients and water spanning 1 m deep in the soil. The results showed that the order of N concentration was leaves > thorns > stems > roots, that the concentration of P in the leaves, thorns, and stems was similar, and that their values were higher than those in the roots. First, the C:N ratios in the leaves and stems were significantly positively correlated with the ratio in roots. The C:N ratios in each organ showed a significant relationship with the soil alkali hydrolyzable nitrogen (SAN) above a depth of 60 cm. In addition to SAN, soil available phosphorus (SAP) and soil organic carbon (SOC) affect the C:N ratio in the roots. Second, the C:P and N:P ratios in aboveground organs showed no correlations with the ratios in roots. The C:P and N:P ratios in the leaves and thorns have no relationship with soil nutrients, while the C:P ratio in roots was influenced by SAN and SOC in all soil layers. Finally, the N:P ratios in roots were also affected by nutrients in different soil depths at 0–20 and 60–80 cm. These results illustrate that the roots were more sensitive to soil nutrients than the aboveground parts. Our study of ecological stoichiometry also suggests a novel systematic approach for analyzing the sensitivity of responses of an organ to environmental conditions.

## Introduction

The stoichiometry characteristics of a given species usually have an optimal range to ensure that it occupies the appropriate niche (Peñuelas et al., 2008; Bradshaw et al., 2012). Based on this characteristic property, the ecological stoichiometric study of species and even ecosystems can reflect the law of species succession and the driving mechanism of environmental factors (Elser et al., 2000; Sterner and Elser, 2002; Ågren, 2008; Yu et al., 2015). However, the stoichiometry characteristics of close relatives or species may vary greatly under different environmental conditions (Yang et al., 2015). These changes are not only the basis of species ecological adaptability but also an important prerequisite for estimating the direction of species evolution under global change conditions (Elser et al., 2010; Yu et al., 2015).

Elemental stoichiometry can reflect important ecological processes, and it is thus considered as a new quantitative analysis approach by ecologists recently (Elser et al., 2000; Sterner and Elser, 2002). Carbon (C), nitrogen (N), and phosphorus (P) are not only the essential factors to construct biology and support their metabolism but also the key carriers of biogeochemical cycle processes, such as nutrient absorption, biological nitrogen fixation, and litter decomposition (Aerts and Chapin, 1999; Elser, 2007). The C:N and C:P ratios are great indicators of the utilization efficiency of N and P under same carbon sequestration conditions (Wang et al., 2015; Zhang et al., 2018a). The C:N and C:P ratios in litter represent the garbage decomposed by microorganisms (Freschet et al., 2012; Hobbie, 2015; Zhang et al., 2018a, 2019a). The N:P ratio is also an important indicator to measure the limit of N or P in the soil (Koerselman and Meuleman, 1996; Luo et al., 2021).

In certain habitats, plants may survive by changing the stoichiometry among their organs. The relationship of the C:N, C:P, and N:P ratios with plant organs, such as leaves, stems, and roots, reflects the biophysical trade-offs of plant resource acquisition and growth strategy (Li et al., 2017; Zhang et al., 2019a; Luo et al., 2021). Leaves are an essential organ for C fixation, and it need much more N and P than stems and roots to perform a high metabolic function. Roots are the major organ for the absorption of N and P, but it always need much more C than leaves to construct their structure. When key nutrient resources in the environment are limited, the decline of the C:N and C:P ratios in stems are more severe than that in leaves (Minden and Kleyer, 2014), indicating that stems may provide some nutrients to leaves. In addition, stem and root cell walls can store large amounts of N (Onoda et al., 2004; Schreeg et al., 2014), which can be reactivated under drought conditions (Chapin et al., 1990). Therefore, stems and roots are not only supporting organs but also the key organs for plants to absorb, transport, and store nutrients (Aoyagi and Kitayama, 2016).

Compared with humid terrestrial areas, the driving effect of environmental variables on plant ecological stoichiometry is more complex in arid areas. We summarized several main reasons described in the following: (a) the available nitrogen in the soil mainly comes from the decomposition of litter by microorganisms, and it decreases gradually when soil depth increases (Jobbágy and Jackson, 2001; Goebes et al., 2019; Guo et al., 2020). Drought can reduce the decomposition of litter by soil microorganisms, leading to nitrogen limitation in arid regions (Kou, 2018). N is considered to be the most important limiting factor in the desert ecosystem. In order to overcome N limitation in the desert ecosystem, legumes are relatively increased to fix N_2_ by biological nitrogen fixation (BNF) (Arndt et al., 2004). At the same time, the C:N ratio of plants increases significantly in arid environments to improve nitrogen use efficiency (Dijkstra et al., 2012; Delgado-Baquerizo et al., 2013). (b) Many studies have shown that vegetation in arid areas often shows P limitation, especially in stems and roots. This limitation might be caused by water deficiency (He et al., 2015). The available phosphorus in the soil comes from the weathering of soil, and it also decreases when soil depth increases (Jobbágy and Jackson, 2001; Guo et al., 2020). Bare lands in arid areas are prone to severe wind erosion and high sun exposure, and their P concentration increases considerably (Delgado-Baquerizo et al., 2013). Li et al. (2010) showed that mean annual precipitation (MAP) is positively correlated with the P concentration in leaves but is negatively correlated with the N:P ratio in arid regions. In contrast, some studies have found that the N and P concentrations of leaves in arid regions are not generally related to MAP (Tao and Zhang, 2015; Zhang et al., 2018b). A meta-analysis by He and Dijkstra (2014) also showed that drought stress has a negative effect on P and a positive effect on the N:P ratio. They also indicated that the availability of water, rather than the availability of P, might be the main driving force for plant stoichiometry under drought stress conditions. (c). Low precipitation seriously limits the infiltration of nutrients, resulting in the enrichment of soil nutrients in the upper soil (Zhang et al., 2019b). However, drought promotes the development of plant roots in-depth to absorb water and nutrients in deeper soil, which makes it difficult to absorb nutrients in the surface layer (Guo et al., 2020). (d) The stoichiometry characteristics of different organs might be influenced by different factors. Luo et al. (2021) found that the ecological stoichiometric characteristics of leaves, stems, and roots of desert plants may be limited by different factors, in which soil factor is dominant and climate factor plays an indirect role (Luo et al., 2021). He et al. (2015) found that the stoichiometry of leaves in a desert plant *Reaumuria soongorica* are influenced by climatic factors, but stoichiometry of stems and roots are mostly affected by soil P. In a nutshell, precipitation in arid regions might affect the distribution of soil nutrients at different depths, and in turn, affects the stoichiometric characteristics of plants.

*Alhagi sparsifolia* is widely distributed in arid areas of Central and Eastern Asia. It has a drought-tolerant potential in desert areas, where it plays an important role in maintaining the stability of the desert ecosystem and preventing desertification (Zeng et al., 2002). *A. sparsifolia* is a leguminous phreatophyte (Zhang et al., 2018a). BNF is an important way to absorb nitrogen, and its developed roots can also absorb nutrients from groundwater (Arndt et al., 2004; Li et al., 2021). Previous studies have revealed that P, rather than C and N, showed a significant relationship with soil N in arid areas (Zhang et al., 2018a, 2021). Zhang et al. (2019a) showed that the C:N ratio was significant convergence among different organs which was closely related to the redistribution of nitrogen among different organs. However, first, whether the C:P and N:P ratios among different organs are convergence or divergence is still unclear. Second, the research on the relationship between nutrients and plants in vertical soil depth is quite scarce. It is still controversial whether soil moisture and nutrients play a significant role in the ecological stoichiometry of deep-rooted plants. To answer these questions, we studied the C:N:P ratios among plant organs, especially including both aboveground and underground organs. Over 3 years of data collection and experiments, we explored the C, N, P, C:N, C:P, and N:P ratios and their relationship as well as their response mechanisms to nutrients and water spanning 1 m deep in the soil. More importantly, we analyzed the associations of plant organ C:N:P stoichiometry with nutrients and soil at different depths by integrated data analysis. Our results will contribute toward a better understanding of arid *Alhagi sparsifolia* plant adaptation to changing environments in Central Asia.

## Materials and Methods

### Study Area and Sampling Sites

**Figure 1:**
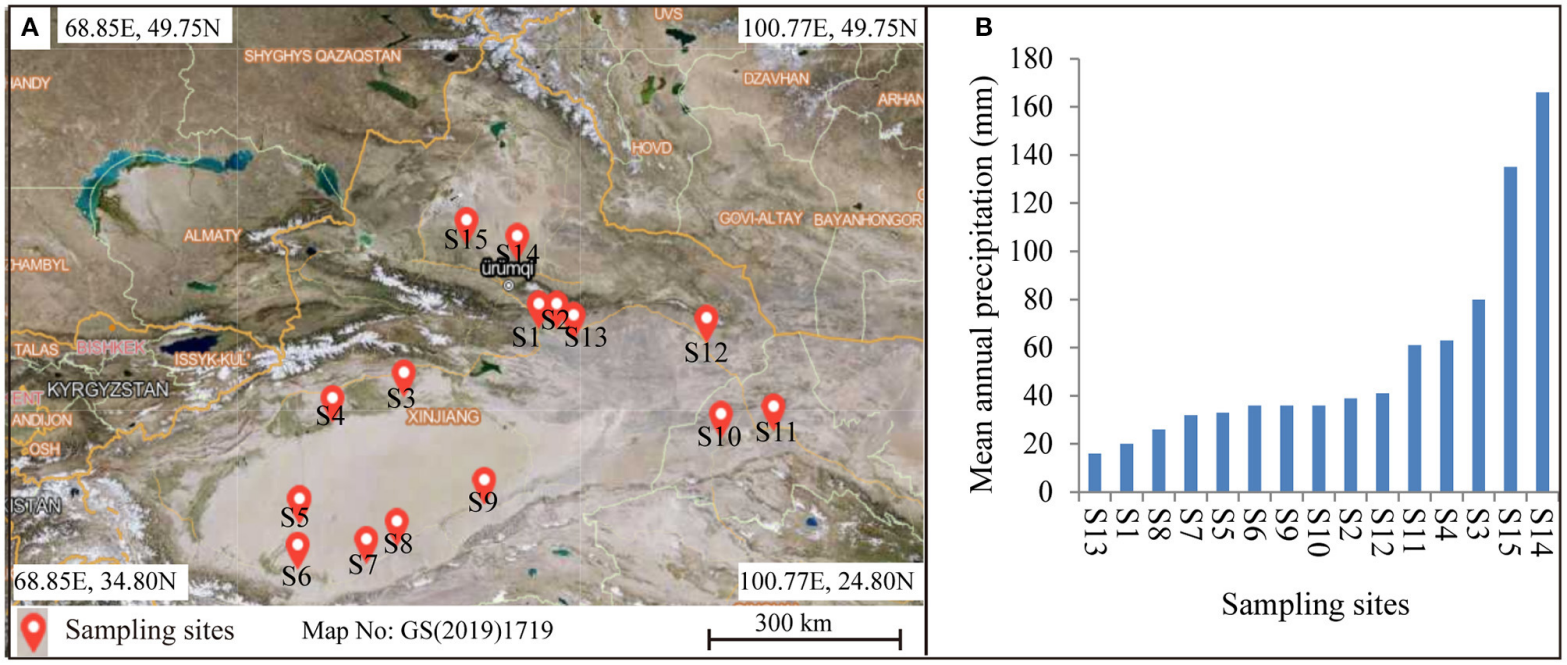
**(A)** Geographical map showing the locations of the sampling sites used in this study, and **(B)** gradient in mean annual precipitation across the distribution of the sites.

*Alhagi sparsifolia* is mainly distributed in the Xinjiang and Gansu provinces in northwestern China. To expand the heterogeneity of environmental conditions, 15 sampling points covered the area of longitude 80.68–96.34°E and latitude 36.85–44.71°N (Figure 1A). The mean annual precipitation (MAP) of the study location ranged from 16 mm to 166 mm were selected for sampling (Figure 1B; Supplementary Table 1). In order to make the data more representative, a sampling point with *A. sparsifolia* as the dominant species was selected. We used the “WorldClim Version 2.0” database to determine precipitation data (with a spatial resolution of ~1 km^2^) (Fick and Hijmans, 2017). At each sampling site, three 10 × 10 m plots were randomly positioned and arranged for sampling.

### Sample Collection and Measurement

Plants were harvested during the growing season (late July to early August) in 2018. At each plot, we randomly chose three individuals of *A. sparsifolia*, which were completely extracted, including their roots above a 100 cm depth. The plant samples were divided into three parts, such as leaves, thorns, stems; roots were dried at 80°C for 48 h, and then ground using a ball mill (Rigaku, Tokyo, Japan) for N and P analyses. Total N was measured with the Kjeldahl method (FOSS Kjeltec 8400, FOSS, Hoganas, Sweden). After digestion with H_2_SO_4_-H_2_O_2_-HF, the ammonium molybdate/stannous chloride method was used for the colorimetric determination of P (Kuo, 1996).

In each plot, we used the three soil profiles formed when digging out the roots to collect soil samples in five depth layers: 0–20, 20–40, 40–60, 60–80, and 80–100 cm. Each component of the soil samples (~100 g) was collected in an aluminum box and then oven-dried to determine soil water content (SWC). Another component of the soil samples, ~500 g was air-dried and passed through a 100-mesh sieve for the subsequent determination of soil alkali hydrolyzable nitrogen (SAN) and soil available phosphorus (SAP). A ball mill (Rigaku, Tokyo, Japan) was used to grind a part of the soil into fine powder, which was used to determine soil organic carbon (SOC). As described by Bao (2000a), the alkaline solution diffusion method was used to evaluate SAN. After extraction with a solution of 0.5 mol/L NaHCO_3_, SAP was evaluated using the molybdate/ascorbic acid blue method (Bao, 2000b). To evaluate SOC, the soil samples were digested in a K_2_Cr_2_O_7_-H_2_SO_4_ solution on a heating plate for 5 min and then measured by titration (Bao, 2000c). The mean values of SAN, SAP, SOC, and SWC across 0–100 cm and the soil classification in each sampling site are listed in Supplementary Table 1.

### Statistical Analyses

We calculated the mean, standard error (SE), and coefficient of variation (CV) of C, N, P, C:N, C:P, and N:P in the leaves, thorns, stems, and roots using all of their values across 45 plots. An ANOVA and the Tukey test were performed to test for differences in the C, N, P, C:N, C:P, and N:P ratios in the leaves, thorns, stems, and root organs, implemented in SPSS 22.0. A reduced major axis (RMA) regression analysis was performed to show the relationships among C, N, and P within the same organ (Yang et al., 2014; Hu et al., 2018), implemented with the “lmodel2” package in R v4.0.2 (R Development Core Team). To assess the relationship of SAN, SAP, SOC, and SWC with MAP, Pearson's correlation analyses were performed on the average data of each plot, implemented using the “vegan” package in R v4.0.2. A linear regression analysis was performed to explore the relationship between SAN, SAP, and SOC with SWC using the “vegan” package in R v4.0.2. Here, Log_10_ transformation was applied to the data to increase the linear relationship. According to MAP and the mean value of SWC across the entire soil profile, a classification was carried out with the system clustering method in SPSS 22.0, which divided the sampling sites into three clusters. ANOVA and the Tukey test were performed to test for differences among the three clusters in terms of MAP, SWC, and C:N:P stoichiometry per organ (done in SPSS 22.0). Their boxplot diagrams were drawn in R v4.0.2. To discern the relationship of C:N, C:P, and N:P of each organ with the SAN, SAP, and SOC in each soil depth, Pearson's correlations were performed on the whole data set using the “vegan” package in R v4.0.2.

## Results

### Concentration and Stoichiometry of C, N, and P Among Organs of *A. sparsifolia*

Among the 15 sampling sites, in each organ, C had a lower CV than N and P. The CV of the C:N ratios among all the organs were similar, while the CV of C:P and N:P in the leaves was significantly lower than that in the stems and roots. The concentration of C was the highest in the thorns and roots (424.2 and 428.9 mg g^−1^, respectively) and the lowest in the leaves (396.2 mg g^−1^). The concentration of N was the highest in the leaves (16.48 mg g^−1^) but the lowest in the stem and root parts (7.91 and 8.93 mg g^−1^, respectively), and the opposite was true for C:N. The concentration of P was similar among the leaf, thorn, and stem organs (1.17, 0.95, and 1.11 mg g^−1^, respectively), all of which exceeded their concentration in root parts (0.56 mg g^−1^). The ratio of C:P was the highest in the roots but the lowest in the leaves. The value of N:P in the leaves was between 14 and 16 but higher in the roots, whereas the value of N:P in the thorns and stems was lower than 14 (Table 1).

**Table 1:** Carbon (C), nitrogen (N), and phosphorus (P) concentrations and the C:N, C:P, and N:P ratios in leaf, thorn, stem, and root organs of *Alhagi sparsifolia*.

	**Leaf**		**Thorn**		**Stem**		**Root**	
	**Mean ± SE**	**CV**	**Mean ± SE**	**CV**	**Mean ± SE**	**CV**	**Mean ± SE**	**CV**
C (mg g^−1^ )	396.2 ± 6.3C	0.11	424.2 ± 5.3BC	0.08	408.0 ± 4.9AB	0.08	428.9 ± 6.0A	0.09
N (mg g^−1^ )	16.48 ± 0.57A	0.23	10.07 ± 0.27B	0.18	7.91 ± 0.24C	0.21	8.93 ± 0.25C	0.19
P (mg g^−1^ )	1.17 ± 0.06A	0.32	0.95 ± 0.06A	0.40	1.11 ± 0.11A	0.70	0.56 ± 0.03B	0.38
C:N	24.81 ± 0.82C	0.22	42.48 ± 1.57B	0.25	53.80 ± 1.74A	0.22	49.86 ± 1.63A	0.22
C:P	368.3 ± 17.9C	0.33	497.2 ± 29.5BC	0.40	536.0 ± 41.6B	0.53	895.3 ± 55.4A	0.42
N:P	14.63 ± 0.34B	0.15	11.60 ± 0.43C	0.25	9.61 ± 0.59C	0.42	17.94 ± 0.9A	0.38

*Mean value (Mean), standard error (SE), coefficient of variation (CV, defined as standard deviation/Mean). Statistically significant differences between organs were determined by ANOVA and the Tukey test using SPSS 22.0, these are indicated by different capital letters*.

**Figure 2:**
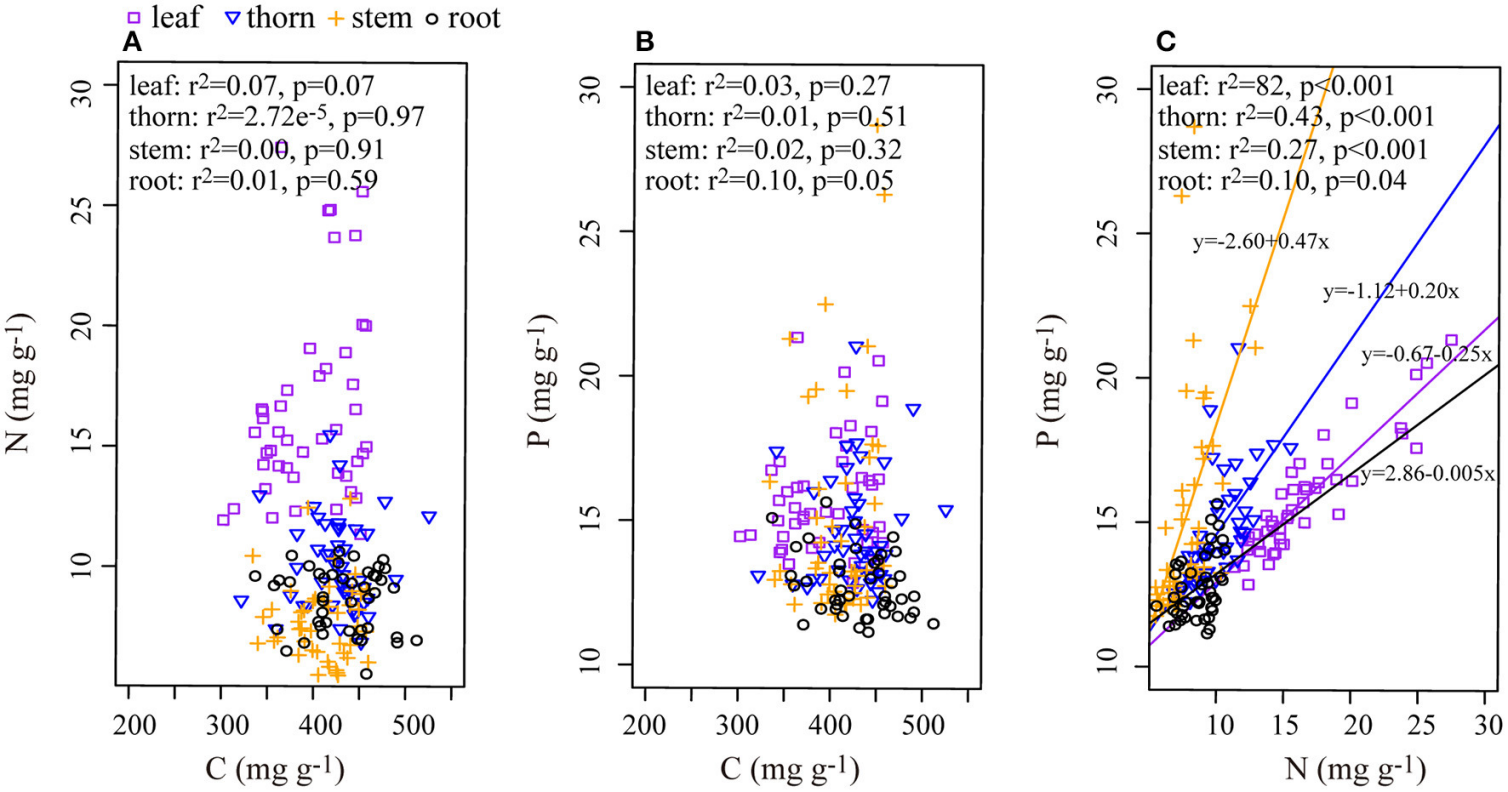
Relationship among C, N, and P in the leaf, thorn, stem, and root organs of *Alhagi sparsifolia*. **(A)** The relationship between C and N; **(B)** the relationship between C and P; **(C)** the relationship between N and P. Regression lines are shown only for relationships that were significant at *p* < 0.05.

In each organ, C and N, as well as C and P, had no apparent relationship (Figures 2A,B), although N and P had positive significant relationships in every organ (Figure 2C). There was a significant positive correlation of C:N between roots and leaves or stems, but the C:P and N:P ratios in the roots did not correlate significantly with those in the aboveground organs (Table 2).

**Table 2:** Correlations of C:N, C:P, and N:P ratios among the leaf, thorn, stem, and root organs of *Alhagi sparsifolia*.

	**C:N_**L**_**	**C:P_**L**_**	**N:P_**L**_**	**C:N_**T**_**	**C:P_**T**_**	**N:P_**T**_**	**C:N_**S**_**	**C:P_**S**_**	**N:P_**S**_**	**C:N_**R**_**	**C:P_**R**_**	**N:P_**R**_**
C:N_L_	1											
C:P_L_	**0.91**	1										
N:P_L_	**0.41**	**0.76**	1									
C:N_T_	0.26	**0.31**	0.26	1								
C:P_T_	**0.42**	**0.60**	**0.64**	**0.82**	1							
N:P_T_	**0.38**	**0.62**	**0.75**	0.05	**0.62**	1						
C:N_S_	**0.65**	**0.69**	**0.49**	0.20	**0.40**	**0.42**	1					
C:P_S_	**0.53**	**0.72**	**0.74**	0.12	**0.53**	**0.75**	**0.62**	1				
N:P_S_	**0.35**	**0.57**	**0.68**	0.06	**0.47**	**0.73**	**0.31**	**0.94**	1			
C:N_R_	**0.44**	**0.46**	**0.31**	−0.14	0.12	**0.39**	**0.32**	**0.33**	0.25	1		
C:P_R_	0.04	0.11	0.17	−0.01	0.12	0.22	0.06	0.07	0.06	**0.53**	1	
N:P_R_	−0.19	−0.12	0.04	0.06	0.08	0.06	−0.10	−0.08	−0.06	0.10	**0.90**	1

*Numbers in bold indicate that the correlation is significant at the 0.05 level (Pearson correlation method, two-tailed)*.

### Effects of MAP and SWC on Soil Nutrients at Different Depths of the Soil Profile

**Figure 3:**
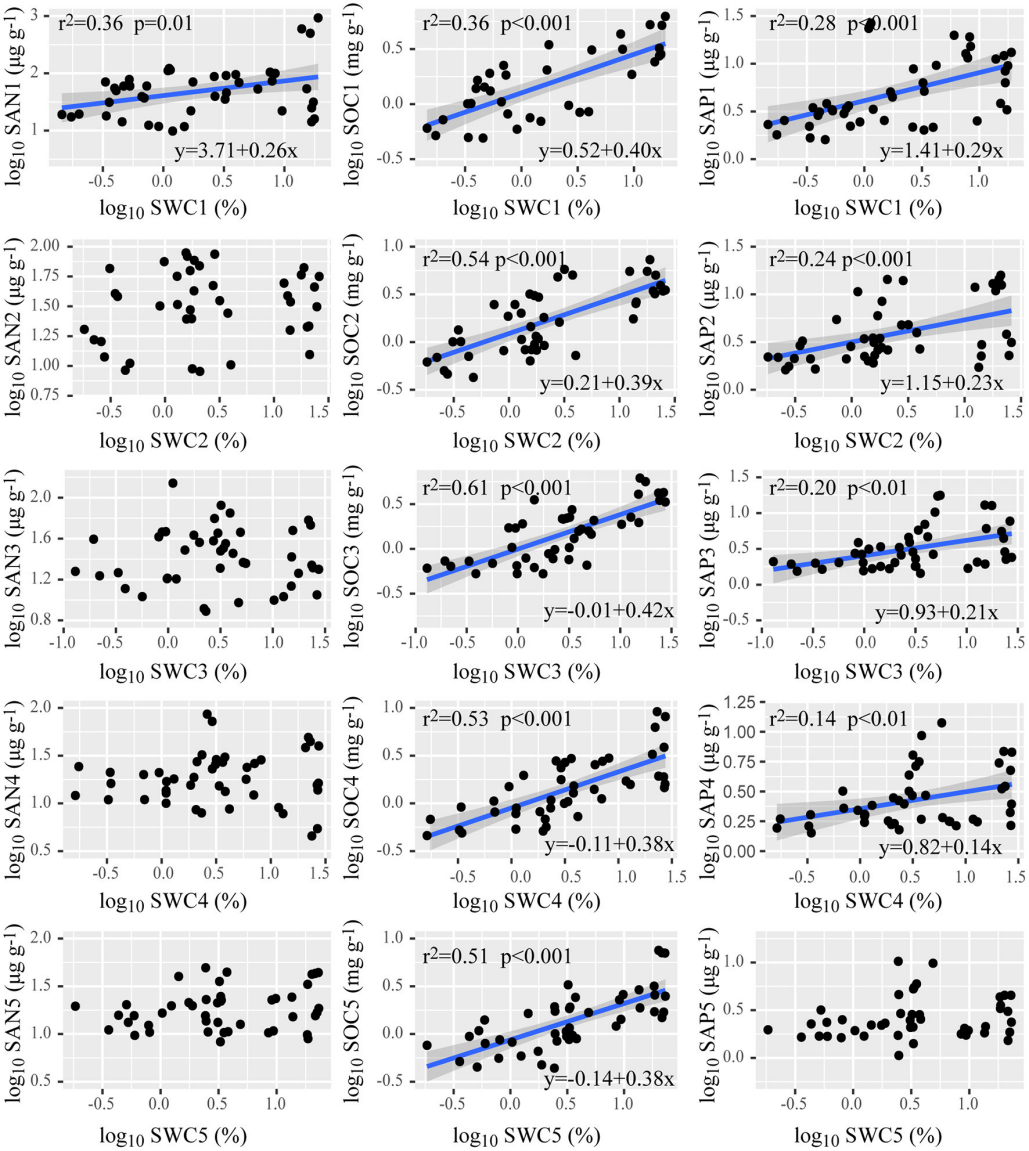
Relationships of soil water content with the available nutrients in the soil at depths of 0–20, 20–40, 40–60, 60–80, and 80–100 cm. SAN1–5 is soil alkali hydrolyzable nitrogen at 0–20, 20–40, 40–60, 60–80, 80–100 cm depths; likewise, for SOC1–5, soil organic carbon; SAP1–5, soil available phosphorus; SWC1–5, soil water content. Regression lines (blue lines) are shown only for relationships that were significant at *p* < 0.05. Shaded bands around each line represent the 95% confidence interval for the linear regression.

There was no significant correlation of SAN, SAP, SOC, and SWC with MAP, across all of the sampling sites (Table 3). SOC in each layer increased with SWC; SWC showed a positive relationship with SAP in the soil above an 80 cm depth, and a positive relationship with SAN above a 20 cm depth (Figure 3).

**Table 3:** Soil nutrients show no significant relationship with mean annual precipitation.

	**MAP**										
	***r***	***p***		***r***	***p***		***r***	***p***		***r***	***p***
SAN1	−0.26	0.36	SAP1	−0.09	0.74	SOC1	0.33	0.23	SWC1	0.36	0.19
SAN2	−0.23	0.41	SAP2	0.04	0.90	SOC2	0.41	0.12	SWC2	0.41	0.13
SAN3	−0.40	0.14	SAP3	0.18	0.52	SOC3	0.22	0.43	SWC3	0.36	0.19
SAN4	−0.33	0.23	SAP4	0.34	0.22	SOC4	0.21	0.46	SWC4	0.31	0.25
SAN5	−0.30	0.28	SAP5	0.37	0.18	SOC5	0.18	0.52	SWC5	0.30	0.27

*MAP, mean annual precipitation; SAN1–5 is soil alkali hydrolysable nitrogen at 0–20, 20–40, 40–60, 60–80, and 80–100 cm depths; likewise, for SOC1–5, soil organic carbon; SAP1–5, soil available phosphorus; SWC1–5, soil water content*.

### Correlation of C:N:P Stoichiometry in Leaf, Thorn, Stem, and Root Organs With Environmental Variation

**Figure 4:**
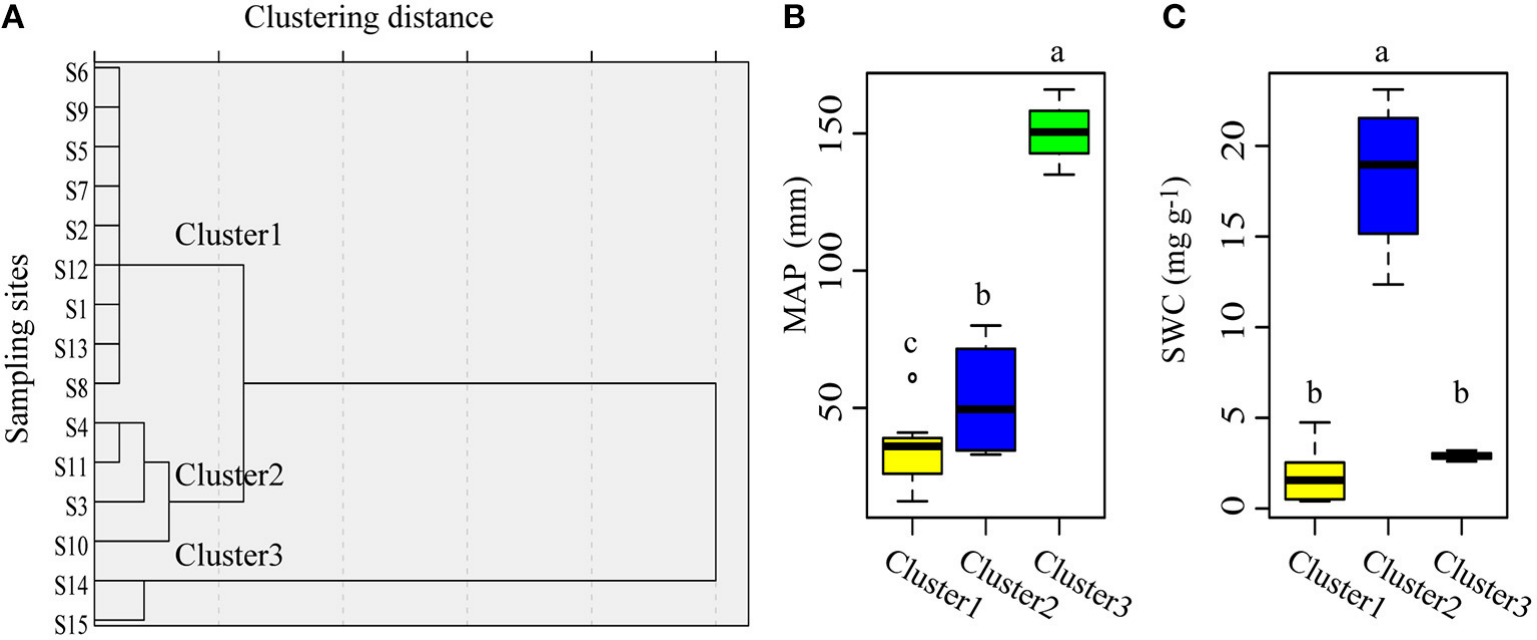
The 15 sampling sites can be classified into three clusters based on their mean annual precipitation and mean soil water content. **(A)** Clustering results of the sampling sites. **(B)** Differences in mean annual precipitation among the three clusters. **(C)** Differences in mean soil water content among the three clusters. S1–S15 denotes sampling sites 1–15.

**Figure 5:**
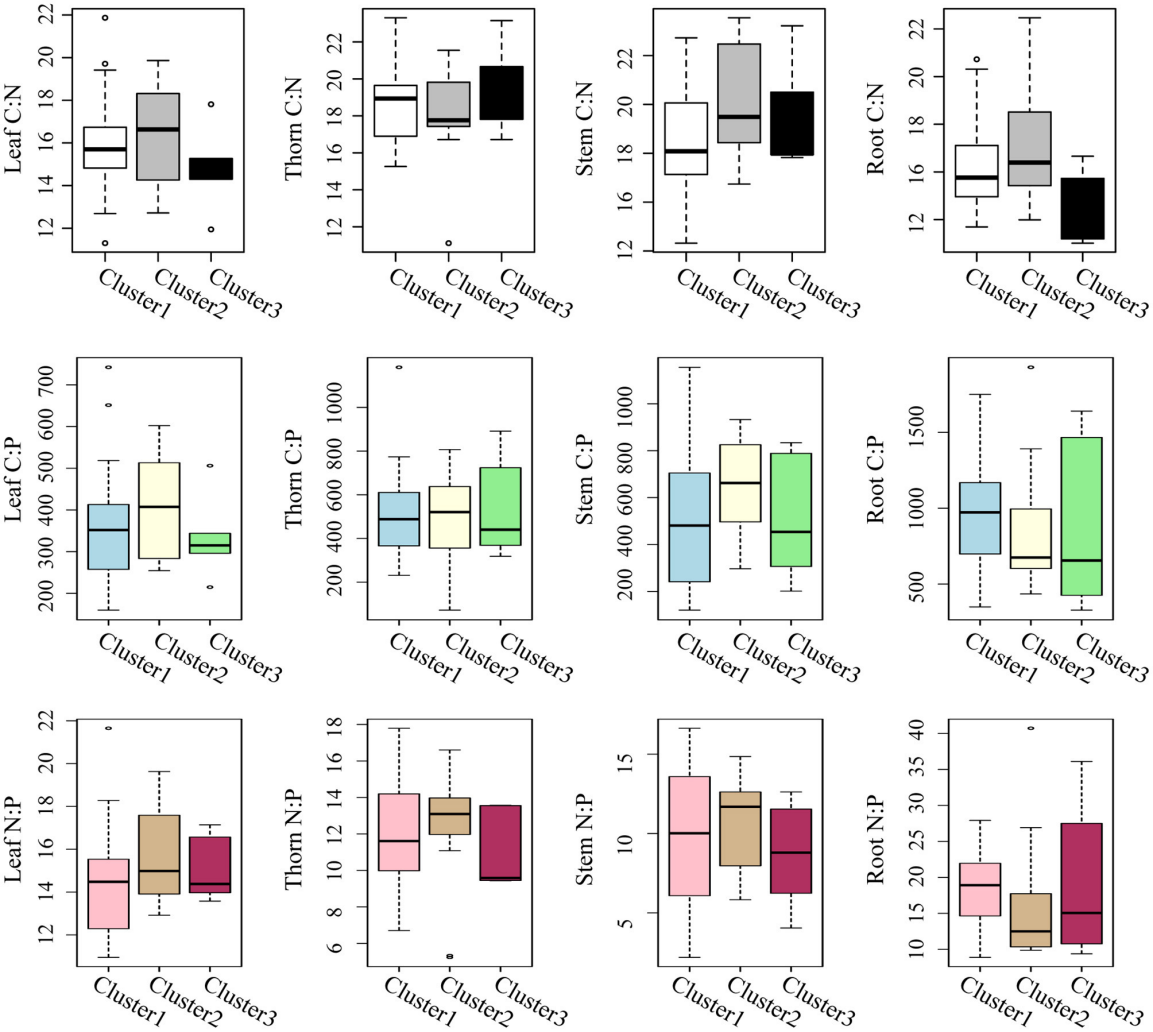
Boxplots of the C:N, C:P, and N:P ratios in leaf, thorn, stem, and root organs of *Alhagi sparsifolia* for the three sampling site clusters that differed in mean annual precipitation and soil water content.

According to their MAP and SWC, the sampling sites could be classified into three clusters (Figure 4A). Cluster1 consisted of sites with low MAP and low SWC; cluster 2 comprised sites with mid MAP and high SWC, while cluster 3 included sites with high MAP and low SWC (Figures 4B,C). For the same plant organ, its C:N, C:P, and N:P did not differ significantly among the three clusters based on MAP and SWC (Figure 5).

**Figure 6:**
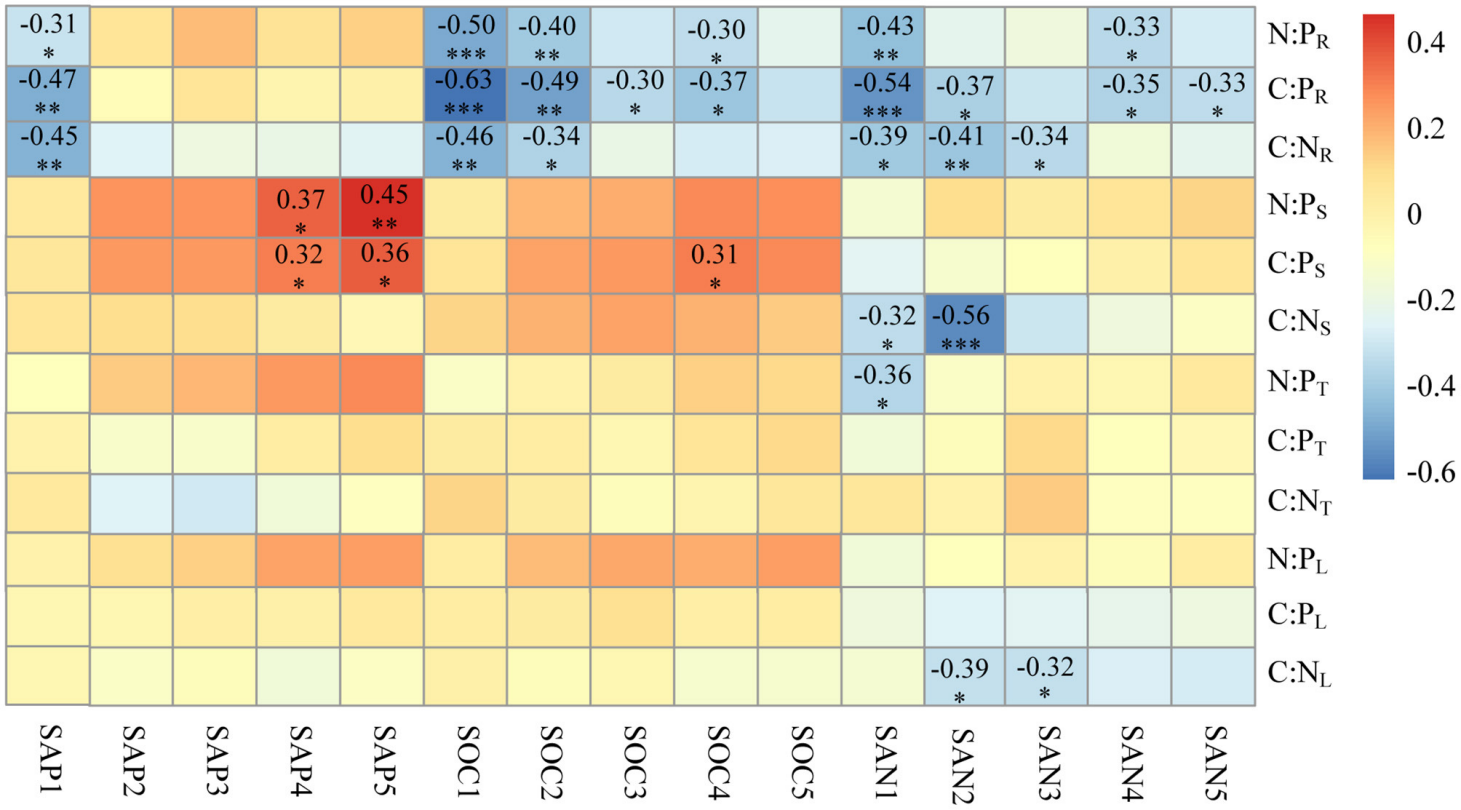
Pearson correlations of the C:N, C:P, and N:P ratios of *Alhagi sparsifolia* with soil nutrients across the 1-m deep soil profile. The numbers shown are the significant correlation coefficients. Statistical significance is denoted by asterisks: ***: *p* <0.001; **: *p* <0.01; *: *p* < 0.05; ns, not significant. The C:N ratios in leaf, thorn, stem, and root organs are abbreviated as C:N_L_, C:N_T_, C:N_S_, and C:N_R_, respectively; likewise for C:P as C:P_L_, C:P_T_, C:P_S_, and for C:P_R_; for N:P as N:P_L_, N:P_T_, N:P_S_, and N:P_R_. SAN1–5 is soil alkali hydrolysable nitrogen at 0–20, 20–40, 40–60, 60–80, 80–100 cm depths; similarly, for SOC1–5, soil organic carbon; SAP1–5, soil available phosphorus.

The C:N ratio of leaves (C:N_L_) and stems (C:N_S_) were negatively affected by the SAN in the soil above 60 cm, especially in the 20–40 cm soil layer (*r* = −0.39 between C:N_L_ and SAN2; *r* = −0.56 between C:N_S_ and SAN2). The N:P (N:P_T_) of thorns was found to be negatively affected by SAN1 (*r* = −0.36). Both the C:P and N:P ratios in stems were positively affected by SAP and SOC in the subsoil layers (below 60 cm), while the C:P and N:P ratios in leaves and thorns had no relationship with soil nutrients. The correlations between soil nutrients and the C:N:P stoichiometry of roots were significantly higher than those of aboveground organs. There was a significant negative correlation between root C:N (C:N_R_) and SAN, and SAP and SOC, above a 60-cm soil depth. Root C:P (C:P_R_) had a negative correlation with SAN and SOC in almost all the soil layers, but correlation weakened with a greater soil depth in the profile. Root C:P also significantly correlated (negatively) with SAP1. Root N:P (N:P_R_) was negatively correlated with SAN1, SAP1, and SOC1 (*r* = −0.43, −0.31, and −0.5, respectively), and SAN4 and SOC4 (*r* = −0.33 and −0.3, respectively) (Figure 6).

## Discussion

For individuals of the same species, the proportion of structural material investment is relatively fixed, so the levels of C should vary less than that of N and P, because both N and P directly affect the metabolic activity of cells (Reich and Oleksyn, 2004; Minden and Kleyer, 2014; Luo et al., 2021). In *A. sparsifolia*, N and P were highest in leaves and lowest in stems and roots, thus demonstrating that the distribution of N and P is consistent with the metabolic functioning of plant organs. However, in line with some herbaceous plants in arid areas (He et al., 2015; Luo et al., 2021), the P in aboveground organs (leaves, thorns, stems) of *A. sparsifolia* are gradually converging, suggesting that all aboveground parts may have a similar growth rate, or rather that all aboveground parts must participate in metabolism together. This may act as compensation for the low photosynthesis rates in leaves. The Leaf N and leaf P concentrations of *A. sparsifolia* were both lower than the corresponding means of terrestrial herbaceous species globally (Han et al., 2005; Tian et al., 2018) leaf N was lower for *A. sparsifolia* than other plant species in typical desertified regions in northern China and Xinjiang (Li et al., 2010; Luo et al., 2021); similarly, its leaf P was lower than that for plants in typical desertified regions in northern China (Li et al., 2010) but similar to that for some desert plants in Xinjiang (Luo et al., 2021). It is worth noticing that the N and P of each organ in different sampling sites showed a great variation, indicating that the same species can show a great response to different environmental conditions. The CV of C:P and N:P ratios of stems and roots were significantly higher than that of leaves, which was consistent with the previous research results, and supported the view that active organs were more stable than inactive organs (He et al., 2015).

As important physiological indexes of plants, the C:N and C:P ratios can reflect the competitive or defensive strategies of species (Sterner and Elser, 2002; Shipley et al., 2006; Rong et al., 2015); moreover, each ratio can also characterize the nutrient use efficiency of plants (Wang et al., 2015). Higher C:N and C:P ratios in stems and roots indicate that these organs are investing much C for support functions. Consistent with plants in nine forest ecosystems in China (Zhang et al., 2019a), the C:N ratio in leaves and stems of *A. sparsifolia* showed a significantly positive relationship with C:N in its roots, and the CV of C:N ratios in each organ was similar. These results indicated that N use strategies of aboveground organs and roots were consistent. In stark contrast, there was no correlation of C:P ratios between the aboveground organs and roots of the plant. Compared with perennial roots, newly generated aboveground organs are distinguished by their rapid growth, which requires more P-rich RNA to sustain protein synthesis (Matzek and Vitousek, 2009). Therefore, compared with roots, the aboveground organs have a lower C:P. Under drought conditions, it may be necessary that more C is invested in various organs of *A. sparsifolia* to construct protective structures; the decoupling of C and N, as well as C and P, is more conducive to adjusting the defense and competition strategies of *A. sparsifolia*.

Leaf N:P is well known as an indicator of soil nutrient limitation for plants (Koerselman and Meuleman, 1996; Drenovsky and Richards, 2004), where N:P <14 signifies N limitation, N:P > 16 implies P limitation, and the case of 14 < N:P <16 signifies N and P co-limitation (Koerselman and Meuleman, 1996). In our study, the leaf N:P of *A. sparsifolia* was between 14 and 16, which suggests that it may be co-limited by N and P, whereas its root N:P was >16. The extremely low concentration of P, and the high C:P and N:P ratios in the roots, together, suggest that P might be a crucial factor that limits the root growth of *A. sparsifolia*, which is common with other N-fixing species. In each organ, N and P showed a significantly positive correlation, and this tightly coupled relationship evinces robust coordination to maintain functioning of this plant in a harsh environment.

### Effects of Mean Annual Precipitation and Soil Water Content on Soil Nutrients at Different Depths of the Soil Profile

Soil alkali hydrolyzable nitrogen and SOC mainly come from the microbial decomposition of plant litter and animal carcasses, while the SAP mainly comes from the wind erosion and desertification of soil, so the nutrients show decreasing trends with soil depth (Goebes et al., 2019; Zhang et al., 2019b; Guo et al., 2020). Precipitation can carry nutrients from the surface layer into the subsoil (Querejeta et al., 2021). However, in most of the sampling sites, MAP was lower than 60 mm and showed no significant relationship with soil nutrients across the soil profile, indicating that lower precipitation might not affect soil nutrients. It is evident that other factors, such as soil texture and topographic position, are more relevant than MAP in water and nutrient availability for plants in the desert. Soil water is another vital hydrological source for plant growth in arid areas. In this study, no significant relationship is shown between SWC and MAP. That is because apart from precipitation, snowmelt is also a driving factor of SWC in arid regions (Meng et al., 2019). The dominant population of *A. sparsifolia* is mainly formed under drought and extreme drought conditions, especially in the extremely arid Turpan Basin, where the MAP is very low, reaching just 16 mm. In the extremely arid southern edge of the Taklimakan Desert, the MAP is only ~36 mm. In such a water-scarce environment, *A. sparsifolia* mainly depends on groundwater or rivers (Zeng et al., 2013).

One of the most important reasons why deep-rooted plants use groundwater in desert areas is the hydraulic lift in their roots (Prieto et al., 2012). There were significant positive correlations of SWC with SOC and SAP, confirming that water availability can directly affect plant nutrient availability in arid areas. However, there was only a positive correlation between SWC and SAN above a 20-cm depth, indicating that SAN in the lower soil depth layers was not affected by SWC, but rather mainly determined by the leaching effect of precipitation on soil nutrients at the surface. Also, roots in soils with higher SWC take up nutrients more actively and have more N-fixing nodules than roots in dryer soils (Li et al., 2021), and then the roots increase soil N content.

### Correlation of C:N:P Stoichiometry in Leaves, Thorns, Stems, and Roots With Environmental Variation

Across the varying MAP and SWC in the arid region, the C:N:P stoichiometry in each organ of *A. sparsifolia* was largely unchanged, indicating that *A. sparsifolia* is quite capable of adjusting to different water conditions in these arid areas and maintaining its homeostasis. In particular, the C:N:P stoichiometry of the aboveground organs of *A. sparsifolia* is more stable than that of its roots. The C:N ratios in leaves, stems, and roots were negatively correlated with SAN in the soil above 60 cm. The significant correlation between plant C:N ratio and SAN may be caused by the following reasons. In terms of N source, *A. sparsifolia* is a leguminous phreatophyte. It can not only fix nitrogen (N_2_) in the atmosphere by BNF, but it can also use nitrate in the groundwater as the main source of nitrogen (Arndt et al., 2004; Li et al., 2021). However, under different groundwater depth conditions, soil nitrogen may still have a great effect on the stoichiometry of C, N, and P in *A. sparsifolia* (Zhang et al., 2018a). We found that SAN1 and SWC were positively correlated and that the SWC in loam can reach about 20%; in this case, soil nitrogen might be an important nitrogen source for *A. sparsifolia* and affect the C:N ratio of *A. sparsifolia*. On the contrary, plant litter is an important source of soil N. Litters with a lower C:N ratio is easier to decompose and release N into the soil. In terms of plant internal, there is a significant relationship between C:N ratios in aboveground organs and that in roots. Roots are not only an important place for BNF but also organs that have direct contact with soil and groundwater. Therefore, they are more closely related to the external environment and can be nutrient-storing tissue. Roots and stems can accumulate more nutrients than necessary to maintain the current metabolic activity and thereafter translocate them to sink tissues like shoot in springtime or leaves when nutrient uptake from the soil is not enough (Zhang et al., 2017). In addition to SAN, SOC1, SOC2, and SAP1 also correlated with the C:N ratio of roots. SOC and SAP could promote plant absorption of N, and also improve BNF of legumes, thus affecting the C:N ratio in roots.

Both the C:P and N:P ratios in stems of *A. sparsifolia* were positively affected by SAP and SOC in the subsoil layers (below 60 cm). The SAP and SOC below 60 cm showed some positive correlation with SWC. Ecological stoichiometry is shaped not only by nutrient uptake but also by plant growth (Aerts and Chapin, 1999). We speculate that under habitat conditions of relatively good water and nutrients, the stem of *A. sparsifolia* may grow well, enabling it to better compete for more light energy, and this increased biomass could have diluted P, resulting in the increased C:P and N:P ratios of stems that we found. The C:P and N:P ratios in leaves and thorns had no relationship with soil nutrients and the C:P and N:P ratios of roots. It is suggested that P concentration of leaves and thorns is mainly distributed on-demand, which is used for metabolism activities to coordinate with N concentration.

The C:P ratio of roots was influenced by SAN1, SAN2, SAN4, SAN5, SOC1, SOC2, SOC3, SOC4, and SAP1, indicating that the C:P ratio of roots was more easily disturbed by external nutrients. Drought could promote soil P transformation and reduces P bioavailability (Zhang et al., 2020). Even across the whole soil profile, SAP increased significantly with an increase in SWC, but only SAP1 could affect the C:P ratio of roots. This finding suggests that the absorption of soil P by deep roots was limited. Relatively sufficient SAN and SOC can promote the absorption of N by plants and enhance their metabolic functioning, thereby presumably enhancing their absorption of P. Accordingly, SAN and SOC in the whole soil profile figure prominently in the determination of the C:P ratio of roots in arid areas. The N:P ratio of roots showed significant negative correlations with SAN1, SAP1, SOC1, SAN4, and SOC4. The results indicated that the nutrients at the soil depths of 0–20 and 60–80 cm have significantly interacted with the roots of *A. sparsifolia*, and that the depth of 60–80 cm corresponds to where its horizontal roots appeared. The metabolic activities of the roots in these **two** layers were significantly stronger, so they showed greater nutrient exchange dynamics with the external environment. In summary, the roots of *A. sparsifolia* are perennial and the aboveground parts are annual; the roots interact with the external environment for a longer time than the aboveground parts, so they should exhibit stronger correlations with environmental variables, and they help in the nutrient homeostasis of leaves, organs with highest metabolic activity.

## Conclusions

The concentration of C was highest in roots and lowest in leaves, while the concentration of N and P was highest in leaves and lowest in stems and roots. It shows that the distribution of nutrients among various organs is compatible with their functions. There was a significant variation in the stoichiometric characteristics of *A. sparsifolia* among the different regions, and the CV of C:P and N: P ratios in stems and roots was significantly higher than that in leaves, indicating that the stoichiometric characteristics of active organs were more stable. Between aboveground organs and roots, C:N coupled well, but neither C:P nor N:P was correlated, which indicated that the ecological stoichiometric of aboveground organs and roots were deferential. The C:N:P stoichiometry in each organ of *A. sparsifolia* remained relatively stable despite differing MAP and SWC in the arid areas, that is because the low MAP and SWC may not play key roles in the growth of *A. sparsifolia*. Soil nutrients showed a significant relationship with the C:N:P stoichiometry of different organs. The interaction of roots with the external environment was significantly more pronounced than that of aboveground organs in *A. sparsifolia*. Roots are places where biological nitrogen fixation occurs and nutrients can be stored, and are in a place where they are in direct contact with soil and groundwater. In addition, the roots of *A. sparsifolia* are perennial, which can provide nutrients for the aboveground parts. However, whether the relationship between the roots and the external environment is related to the growth rate needs to be studied further. In summary, the research on plant ecological stoichiometry should focus not only on leaves but also on roots.

## Data Availability Statement

The original contributions presented in the study are included in the article/Supplementary Material, further inquiries can be directed to the corresponding authors.

## Author Contributions

HY, FZ, and GL designed the study and the experiment. HY carried out field and experimental work, while BZ and AT helped in data analysis. AT, HZ, CG, and FZ contributed to the revision of the draft. All authors contributed to the article and approved the submitted version.

## Funding

This study was financially supported by the Joint Funds of the National Natural Science Foundation of China and the Government of Xinjiang Uygur Autonomous Region of China (U1903102); the National Natural Science Foundation of China (41977050 and 41877012); the team project of the Chinese Academy of Sciences (2018-YDYLTD-002); and the Western Young Scholar Program-B of the Chinese Academy of Sciences (2018-XBQNXZB-018).

## Conflict of Interest

The authors declare that the research was conducted in the absence of any commercial or financial relationships that could be construed as a potential conflict of interest.

## Publisher's Note

All claims expressed in this article are solely those of the authors and do not necessarily represent those of their affiliated organizations, or those of the publisher, the editors and the reviewers. Any product that may be evaluated in this article, or claim that may be made by its manufacturer, is not guaranteed or endorsed by the publisher.
