# Supplementary material for: Stoichiometry of C:N:P in the Roots of Alhagi sparsifolia Is More Sensitive to Soil Nutrients Than Aboveground Organs
Source: Front Plant Sci. 2021 Oct 12;12:698961. doi: 10.3389/fpls.2021.698961 (PMC8545904; doi:10.3389/fpls.2021.698961)
Supplement: Supplementary file 1 [file Table_1.docx]

Table S1. Mean value and standard error of soil characteristics and classification, and climatic data in 15 sampling sites.

| Sampling sites | Classification | SAN  (μg g^-1^) | SAP  (μg g^-1^) | SOC  (mg g^-1^) | SWC  (%) | MAP (mm) |
| --- | --- | --- | --- | --- | --- | --- |
| S1 | Sandy soil | 71.61±13.08 | 3.13±0.23 | 2.22±0.12 | 1.56±0.42 | 20 |
| S2 | Clay soil | 62.29±17.39 | 7.51±4.44 | 4.12±1.47 | 4.75±1.84 | 39 |
| S3 | Loam | 24.38±6.09 | 2.22±0.26 | 3.41±0.81 | 23.11±1.82 | 80 |
| S4 | Loam | 20.84±5.25 | 2.15±0.06 | 1.94±0.19 | 12.36±0.76 | 63 |
| S5 | Sandy soil | 16.26±0.40 | 2.77±0.12 | 0.73±0.03 | 0.46±0.08 | 33 |
| S6 | Sandy soil | 35.96±7.69 | 1.95±0.07 | 1.15±0.01 | 0.51±0.06 | 36 |
| S7 | Sandy soil | 51.27±16.63 | 2.20±0.23 | 0.73±0.05 | 2.19±0.32 | 32 |
| S8 | Sandy soil | 25.76±7.15 | 1.63±0.07 | 0.83±0.12 | 1.19±0.20 | 26 |
| S9 | Sandy soil | 14.48±0.70 | 8.36±1.98 | 0.51±0.02 | 0.40±0.06 | 36 |
| S10 | Loam | 16.92±0.76 | 9.00±2.64 | 3.55±0.37 | 19.97±1.13 | 36 |
| S11 | Loam | 56.67±7.28 | 11.37±1.23 | 7.11±0.30 | 17.95±2.89 | 61 |
| S12 | Clay soil | 51.96±18.07 | 2.94±0.48 | 2.01±0.39 | 4.36±1.71 | 41 |
| S13 | Clay soil | 34.21±4.42 | 4.35±1.96 | 1.46±0.28 | 2.54±0.93 | 16 |
| S14 | Clay soil | 31.26±3.58 | 5.37±0.38 | 3.96±1.19 | 3.20±0.07 | 166 |
| S15 | Sandy soil | 9.47±0.49 | 2.77±0.07 | 0.79±0.03 | 2.60±0.36 | 135 |

Note: Values were Mean value ± standard error across 0–100cm depths in each sampling sites; SAN: soil alkali hydrolysable nitrogen; SOC: soil organic carbon; SAP, soil available phosphorus; SWC, soil water content.
